# Identification of Influential Modules Considering Design Change Impacts Based on Parallel Breadth-First Search and Bat Algorithm

**DOI:** 10.3389/fbioe.2021.791566

**Published:** 2022-01-05

**Authors:** Xianfu Cheng, Zhihu Guo, Xiaotian Ma, Tian Yuan

**Affiliations:** Key Laboratory of Conveyance and Equipment, East China Jiaotong University, Nanchang, China

**Keywords:** modular product, engineering change, change impact degree, influential module, parallel breadth-first algorithm

## Abstract

Modular design is a widely used strategy that meets diverse customer requirements. Close relationships exist between parts inside a module and loose linkages between modules in the modular products. A change of one part or module may cause changes of other parts or modules, which in turn propagate through a product. This paper aims to present an approach to analyze the associations and change impacts between modules and identify influential modules in modular product design. The proposed framework explores all possible change propagation paths (CPPs), and measures change impact degrees between modules. In this article, a design structure matrix (DSM) is used to express dependence relationships between parts, and change propagation trees of affected parts within module are constructed. The influence of the affected part in the corresponding module is also analyzed, and a reachable matrix is employed to determine reachable parts of change propagation. The parallel breadth-first algorithm is used to search propagation paths. The influential modules are identified according to their comprehensive change impact degrees that are computed by the bat algorithm. Finally, a case study on the grab illustrates the impacts of design change in modular products.

## Introduction

The product modularization has a significant influence on the product development process and on the whole product life cycle (Bonvoisin et al., 2016). The modular design has become a widely used and researched product and system development strategy that meets diverse customer requirements. A module is a relatively independent chunk of a system loosely coupled to the rest of the system (Höltt-Otto et al., 2012). There are many methods for designing modular products that can be classified into two main groups: function-based and matrix-based methods (Eppinger and Browning, 2012; Kashkoush and EIMaraghy, 2017). Modular design on the basis of Design Structure Matrix (DSM) has been widely studied in the literature (AlGeddawy and ElMaraghy, 2013; Borjesson and Hölttä-Otto, 2014; Cheng et al., 2018). In modular product architecture, independent complete modules are rare within a given product architecture. There often exists association relationships between modules. Due to various factors, such as changing requirements, improving the environment, technology infusion, and product upgrade, the product architecture may be modified to meet new customer requirements. A change to one module of a product may trigger a series of changes in other modules. To reduce the cost and negative impact of design change, the designers should try to control the change of modules in a product, especially influential modules. It is crucial for designers to decrease the influence of design change by reducing the change of influential modules in product design. Further, the identification of influential modules can help prevent the products failure and distribute the development resources. Whereby, the relative importance or influence of modules is measured in terms of design change impact.

Engineering change is unavoidable during the product evolution process. Manufacturing enterprises sometimes utilize engineering change to deal with technology obsolescence or improve quality with shorter turnarounds. Design change in modular products considers not only the change of module, but also the change of characteristic parameter or assembly constraints of the components in modules. So, the problem of module change can be classified into two types: change of a part propagating to other parts within the same module, and change of a part propagating to other parts in different modules (Lee et al., 2010). Some tools and methods for assessing or predicting the influence of engineering changes on products have been proposed in extant literature (Ollinger and Stahovich, 2004; Yang and Duan, 2012; Ahmad et al., 2013; Hamraz and Clarkson, 2015). However, they tend to be confined to an integrated product architecture or a single product instance (Ollinger and Stahovich, 2004; Lee et al., 2010; Yang and Duan, 2012; Ahmad et al., 2013; Hamraz and Clarkson, 2015; Hein et al., 2021). This paper aims at presenting an approach to analyzing the associations and change impacts between modules in modular products based on design structure matrix and change propagation network. According to the relationships between parts within a module and their change propagation characteristics, the propagation trees of change impacts are constructed, and all possible propagation paths of module change are captured. To effectively manage design change and prevent product failure in modular products, the impacts of design change between modules are analyzed, and the influential modules are identified from the perspective of engineering change. For the sake of simplicity, we will use the term change impact degree to refer to the relative impacts for a part or module being changed on another part or module.

The remainder of the paper is structured as follows: the following section reviews the related research; *Analysis on Change Impact Degree Between Modules* discusses the association and change impact degrees between modules; *Change Propagation Analysis Within Affected Module* identifies reachable parts affected by change propagation and analyzes the change impact degree of the affected part within module; the change influence of affected parts within module is analyzed, and influential modules are identified in *Identification of Influential Modules Considering Design Change Impacts*; next, *Case Study* discusses a case study to illustrate the proposed approach; finally, the conclusion and discussion are remarked in *Conclusion*.

## Related Literature

Engineering change is sometimes an effective evolution mode for enhancing product quality. It enables the product to fulfill affluent market segments, but at the same time, increase product design complexity. If properly managed, changes can provide opportunities for improvement to the product and increase its consumer value (Hein et al., 2021). A change may encompass any modification of the product as a whole or in part, and may alter the interactions and dependencies of the constituent elements of the product (Jarratt et al., 2011). Jarratt et al. (2011) summarized a categorized overview on engineering change and analyzed the nature of the engineering change process. A conprehensive review of engineering change is not repeated here. This section aims to discuss some relevant work from the association representation based on DSM and change propagation between modules and parts in the modules.

Clarkson et al. (2004) and Eckert et al. (2004) used likelihood and impact of change to predict the risk of change propagation, traced potential propagation paths among parts based on DSM, and outlined the methods to compute the risk of direct and indirect change propagation. Giffin et al. (2009) proposed a network-based analysis method using a combination of graph theory and DSM. They developed a set of indices to quantify the relative strength of each area in terms of its propensity. Koh et al. (2012) presented a modeling method matrix-based to assess the effects of engineering change propagation, which set up the house of quality and the change prediction method to model the performance of different change options. Pasqual and deWeck (2012) presented a multilayer network model of change propagation comprising product layer, change layer, and social layer. They developed the repository that includes a few novel tools and metrics for the analysis and management of change propagation. Mehta et al. (2013) proposed an approach to determine important attributes of engineering change, which employed the observed distribution and the domain knowledge to evaluate the importance of a feature set through retrieving similar past engineering changes. Maier et al. (2014) developed a discrete-event simulation model to evaluate the effects of different priority policies, which was based on a product architecture DSM and considered the combined effects of progressive iteration, rework and change propagation. Xie and Ma (2016) presented a graph-based association model to represent the network of constraints, and developed a dynamic inter-feature association map to analyze engineering change propagation. However, this previous research focused heavily on change propagation prediction and its management in an integrated or a single product.

A method based on matrix technique using engineering change forecast to prioritize product parts for modularization was introduced by (Koh et al., 2015), which can be applied to support decision makers in their modularization efforts from a change perspective. Lee et al. (2010) employed the analytic network process to measure design change impacts in modular products, and identified the final priorities of parts with their relative change impacts on the whole product. Ullah et al. (Hein et al., 2021) analyzed effective change propagation quantitative risk-based in a product family design, and adopted a seven-step mechanism comprising of a mathematical model and an algorithm. This approach takes into account direct and indirect change propagation, but does not analyze change impact degree between modules. Cheng et al. (2018b) discussed the coupling between modules in product family design, and presented a method for coupling analysis between platform/customization modules and decoupling strategies. In this method, the coupling between platform modules, between platform module and customization module, as well as between customization modules is depicted, respectively. Cheng et al. (2020) analyzed coupling association problems in design process of modular product design, calculated association dependence degree between modules, as well as addressed corresponding decoupling strategies for coupling modules. Unfortunately, these two proposed methods only consider direct influences of change propagation between two modules, and do not analyze all possible CPPs.

Identifying influential nodes in complex networks has attracted increasing attention in recent years. It is well known that many mechanisms such as spreading, cascading, and synchronizing are highly affected by a tiny fraction of key nodes in complex networks (Liu et al., 2016). Identifying the most efficient “spreaders” in complex network is a crucial step to optimize the use of available resources and ensure the more efficient spread of information (Kitsak et al., 2010). Gao et al. (2013) proposed a method of identifying influential nodes by semi-local centrality combined with modified evidential centrality, which considered the degree distribution to build the basic probability assignment of each node. Zhang et al. (2013) utilized the information transfer probability between any pair of nodes and the k-medoid clustering algorithm to identify influential nodes. Bae and Kim (2014) proposed a ranking method to estimate the spreading influence of a node in complex network using coreness centrality. Liu et al. (2016) proposed a method to evaluate the importance of nodes in complex networks based on degree and the importance of lines, which only needs the local information of nodes. Ahmad et al. (2013) presented an improved cluster rank approach to find influential nodes, which took into account common hierarchy of nodes and their neighborhood set. All of these above focused on influential nodes in a network, and did not discuss the importance of modules as well as the impact of node’s change.

In addition, a few papers available today address influential modules. Specifically, Lee et al. (2010) measured the relative importance of module with design change impacts in modular products through pairwise comparisons. The presented method simplifies tedious works for identifying indirect influences. The main limitation is that it cannot measure the impact of changes in a part or a module on a single part or module. Li et al. (2021) presented a method for function module partition of complex products and systems through community detection using weighted and directed complex networks. Li et al. (2019) identified the influential function modules based on weighted LeaderRank algorithm, and used susceptible-infected-recovered (SIR) model to assess the influence degrees of the identified function modules. However, they thought only of the number of relation types between the modules as the relationship between the modules, and did not consider design change impact. Influential modules could be identified and regarded as design preferences. Above preferences are provided for the designers to manage design change in modular product design. So, in view of the above, this article proposes a quantitative analysis approach that can be employed to assess the importance of modules considering change propagation across different modules.

## Analysis on Change Impact Degree Between Modules

DSM is a popular technique based on a square matrix with identical row and column labels to represent and analyze connections among elements within a system, process, task, or product in a compact, visual, and analytically advantageous format (Steward, 1981; Browning, 2001). DSM has been widely used to support not only modularization for a product or product family but also engineering change (Clarkson et al., 2004; Samling and de Weck, 2007; Hong and Park, 2014; Cheng et al., 2018b).

According to space, material, energy, and information link between parts, the initial DSM of the product are built. The clustering algorithm is introduced to group these parts into modules and to identify interactions between modules. Detailed discussion on DSM to facilitate module identification is not within the scope of this article. The interactions between parts in the DSM can be represented in terms of risk (Clarkson et al., 2004), difficulty (Hoelttae and Otto, 2005), probability of change (Sharman and Yassine, 2017) or dependence (Jung et al., 2018), which would result in rework. Al Handawi (Alhandawi et al., 2020) defined three more changeability aspects based on the nature of change effects, namely, robustness, scalability and modifiability. From the perspective of design change, the change considers heavily the impact of the modification to one part on other parts. This paper aims to analyze relative change impact degree between modules. So, without loss of generality the probability of change propagation is utilized in this work. The probability of change propagation refers to the probability of a redesign of the dependent part being necessary given that a change has occurred in the feeding part (Sharman and Yassine, 2017).

Assume a product consists of a total of *n* parts and is divided into *N* modules, where *C*_*i*_ (*i* = 1, 2, … , *n*) is the *i*th part. These parts are arranged into DSM in the form of modules. Then the influence of design change of one part on another directly connected part, namely the probability of change propagation, is determined through analyzing relevant dependences between them. The new matrix with the probability of change propagation is called design dependency matrix (DDM) of modules.

**FIGURE 1:**
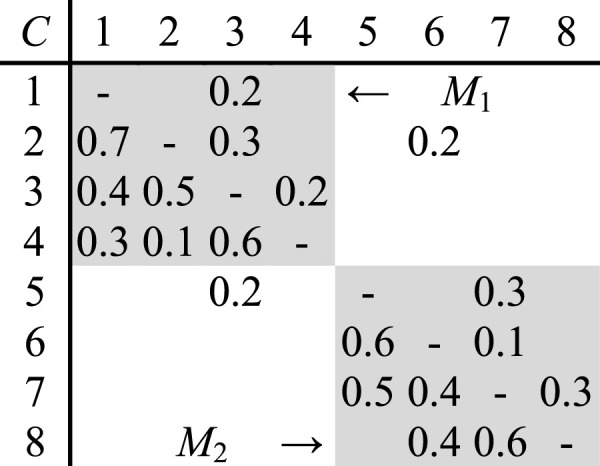
Design dependency matrix with two modules.

For simplicity of explanation on design change impacts between modules, a product comprising of two modules *M*_1_ and *M*_2_, are taken as an example to describe and analyze their relationship and change propagation, as shown in Figure 1. Here, the row and column headings represent parts. The values of off-diagonal elements denote direct dependence between parts, and the direction of change propagation is from the element in the corresponding column to the element in the corresponding row. For instance, the matrix cell “0.5” in the third row and second column, represents the probability of a redesign of part *C*_3_ being necessary given that a change has occurred in part *C*_2_. The modules in DDM interact through bottleneck interactions. An interaction in DDM is considered a bottleneck when it does not allow the decomposition of DSM into mutually separable groups, and therefore appears outside the clusters. Each bottleneck in DDM represents an interaction between modules. There are two interfaces between module *M*_1_ and module *M*_2_. Module *M*_1_ influences part *C*_5_ of module *M*_2_ through part *C*_3_, and part *C*_6_ of module *M*_2_ influences part *C*_2_ of module *M*_1_ specifically. The modification of module *M*_1_ will lead to change of module *M*_2_ and vice versa.

**FIGURE 2:**
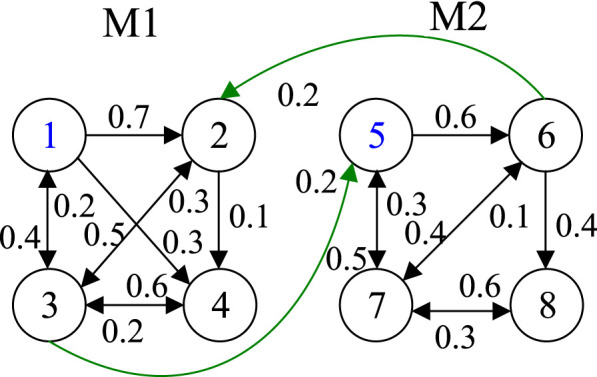
Change propagation network between module *M*_1_ and module *M*_2_.

Since change propagation network can capture all of the direct and indirect impacts among elements, the phenomenon of change propagation in DDM can be effectively mirrored in the network (Lee et al., 2010). The change propagation network between module *M*_1_ and module *M*_2_ corresponding to Figure 1 is shown as Figure 2. Here, the circle represents the node that indicates the product part, the number inside the circle denotes its serial number, an arrow indicates change direction from the part the arrow leaves to that the arrow enters, and the value near the line with an arrow is the corresponding change probability propagated from predecessor to successor.

It can be seen in Figure 2 that the change impact of one module on another module is related not only to the interfaces between modules but also to the association among parts within downstream (affected) module. Assume there is an interaction between module *M*_*p*_ and module *M*_*q*_, where part *C*_*i*_ in module *M*_*p*_ has effect on part *C*_*j*_ in module *M*_*q*_, *C*_*i*_ and *C*_*j*_ are respectively called instigating part and affected part of change propagation from *M*_*p*_ to *M*_*q*_. Since *M*_*q*_ is a “coupling subsystem,” *C*_*i*_ has influence not only on *C*_*j*_ but also on other parts in *M*_*q*_. *C*_*j*_ may be regarded as a feeding part of change propagation within *M*_*q*_. It can directly and indirectly affect other parts in *M*_*q*_. Therefore, there is a need to analyze direct and indirect change impacts of *C*_*i*_ on *M*_*q*_ and to capture its change propagation paths. Simultaneously the change impact degree of *C*_*j*_ on other parts within *M*_*q*_ should also be computed.

Assume *M*_*q*_ consists of *m* parts, where *m*_*q*_ and *n*_*q*_ are the index of the first part and the last part of *M*_*q*_ in DDM, respectively. The change impact degree propagated from *C*_*j*_ to the *k*th part *C*_*k*_ in *M*_*q*_ is defined as *P*(*C*_*j*_, *C*_*k*_) (*k* = *m*_*q*_, *m*_*q*_+1, … , *n*_*q*_, *k*≠*j*), then the change impact degree of *C*_*i*_ on *M*_*q*_, denoted as *P*(*C*_*i*_, *M*_*q*_), can be defined as follows.

$$P\left(C_{i},M_{q}\right)=r_{j,i}\cdot \left[1+\sum_{k=m_{q},k\neq j}^{n_{q}}P\left(C_{j},C_{k}\right)\right]$$ (1)

where, *r*_*j*,*i*_ is the probability of change propagation (direct change impact) of *C*_*i*_ in *M*_*p*_ on *C*_*j*_ in *M*_*q*_, and $r_{j,i}\cdot \sum_{\begin{array}{l} k=m_{q}\\ k\neq j \end{array}}^{n_{q}}P\left(C_{j},C_{k}\right)$ is indirect change impacts of *C*_*i*_ on other parts in *M*_*q*_.

If *C*_*i*_ affects directly more than one part in *M*_*q*_, for example, *C*_*i*_ affects both parts *C*_*u*_ and *C*_*v*_ in *M*_*q*_, then *P*(*C*_*i*_, *M*_*q*_) should be the sum of their change impact degree, expressed as in Eq. (2).

$$P\left(C_{i},M_{q}\right)=r_{u,i}\cdot \left[1+\sum_{\begin{array}{l} k=m_{q}\\ k\neq u \end{array}}^{n_{q}}P\left(C_{u},C_{k}\right)\right]+r_{v,i}\cdot \left[1+\sum_{\begin{array}{l} w=m_{q}\\ w\neq v \end{array}}^{n_{q}}P\left(C_{v},C_{w}\right)\right]$$ (2)

where, *r*_*u*,*i*_ and *r*_*v*,*i*_ are respectively the probability of change propagation of *C*_*i*_ on *C*_*u*_ and *C*_*v*_, *P*(*C*_*j*_, *C*_*k*_) and *P*(*C*_*j*_, *C*_*k*_) are respectively change impact degree of *C*_*u*_ and *C*_*v*_ on *C*_*k*_ in *M*_*q*_.

The change impact degree propagated from upstream module to downstream module can be measured by computing the total change impact degree of all instigating parts. If *M*_*p*_ has *t* instigating parts that have influence on *M*_*q*_, then the total change impact degree, *P*(*C*_*i*_, *M*_*q*_) propagated from *M*_*p*_ to *M*_*q*_, can be represented as

$$P\left(M_{p},M_{q}\right)=\sum_{i=1}^{t}P\left(C_{i},M_{q}\right)$$ (3)

## Change Propagation Analysis Within Affected Module

The change between modules is related to the association among parts within an affected module and the influence of the affected part in the corresponding module in which it is located. The larger the size of the module and the stronger the influence of the affected part in the corresponding module, the greater the scope of change propagation and the more the change propagation paths.

### Identify Reachable Parts for Change Propagation

Before analyzing CPPs, it is necessary to identify which parts are influenced directly or indirectly by the affected part within the corresponding module in which it is located. DDM only represents direct dependence relationships between parts, and does not directly reflect reachable parts of change propagation of affected part. In this paper, reachable matrix is employed to achieve this task. According to DDM with clustered modules, it is possible to determine whether there are dependencies between any two modules. The modules in which affected parts are located are extracted as sub DDM. Then all of the non 0 value in the cells are modified to the value “1”. Its reachable matrix is computed, and the parts influenced directly and indirectly by affected part are judged. Here the affected part is also regarded as a feeding part of change propagation in the corresponding module. These influenced parts are reachable parts of change propagation. In reachable matrix, if there is one or more cell “0” in the column in which feeding part is located, it means that the part corresponding to this element does not rely on feeding part, and then the row and column in which this part is located can be deleted. Now, the matrix after dimension reduction is called the reduction matrix of reachable matrix.

**FIGURE 3:**
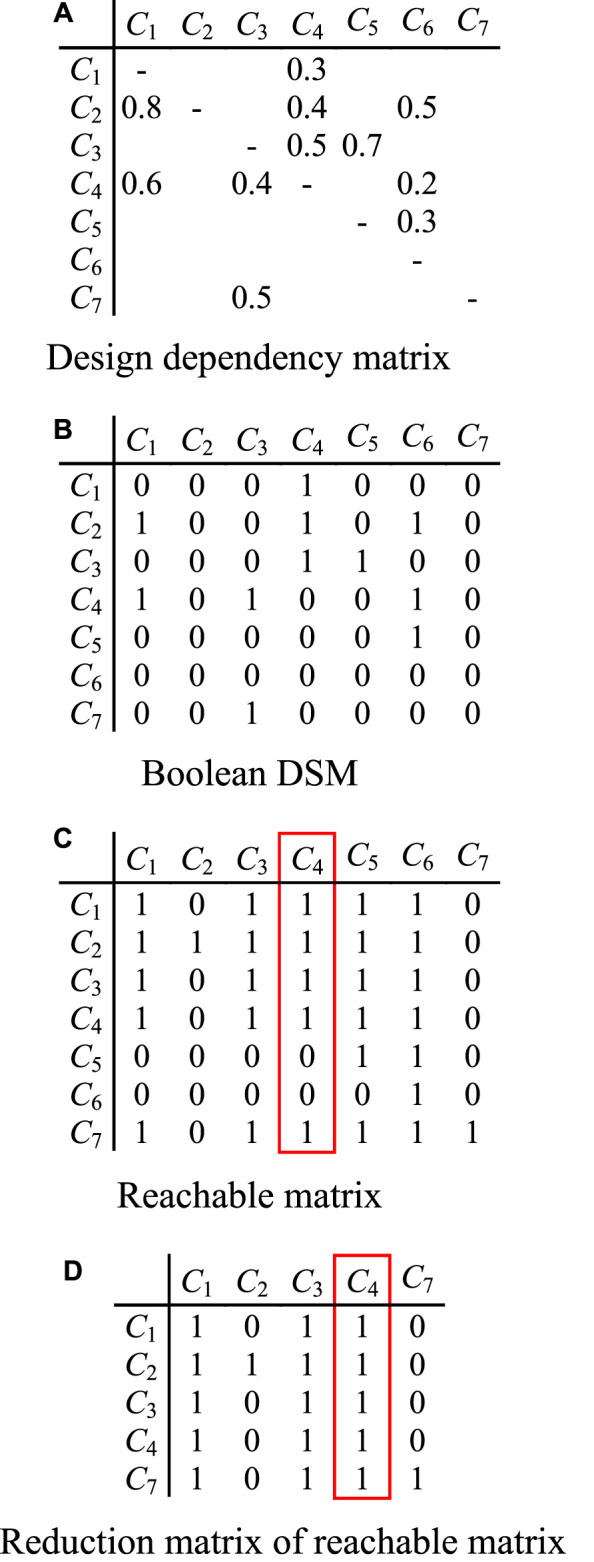
Identifying reachable parts of change propagation using reachable matrix.

Let us consider a DDM of a module comprising seven parts, as shown in Figure 3A. Assume part *C*_4_ is an affected part propagated from another module. Here, *C*_4_ can be taken as a feeding part of change propagation. Then initial design dependency matrix should be converted to Boolean DSM and reachable matrix, as shown in Figures 3B,C.

It is evident from Figures 3A,B,C, that *C*_4_ passes the change onto the directly connected parts *C*_1_, *C*_2_, and *C*_3_ (they may be also indirectly influenced), and indirectly influences part *C*_7_ through intermediate parts. Hence, *C*_1_, *C*_2_, *C*_3_, and *C*_7_ are all reachable parts of change propagation from *C*_4_. In addition, the alteration of *C*_4_ does not propagate to parts *C*_5_ and *C*_6_. Here, reachable matrix can be reduced, as shown in Figure 3D. So, during analyzing all possible paths of change propagation of part *C*_4_, parts *C*_5_ and *C*_6_ can be ignored.

### Search Change Propagation Paths Based on Parallel Breadth-First Algorithm

This section represents an intelligent parallel breadth-first algorithm to search change propagation paths based on the design dependency matrix and directed graph. Its basic idea is: instigating part of the module is regarded as start vertex *V*_0_ of directed graph, the adjacent vertex (parts) directly associated with *V*_0_ are accessed; then they will be taken as new vertices in turn, and the vertices that has not been accessed and is directly related to them are continuously accessed. Repeat the process until access traverses all the vertices.

The change propagation network can be represented as a directed graph, *G=*<*V*, *E* >, wherein the elements of *V* = (*v*_1_ *, v*_2_ *,…,v*_*n*_) are the nodes (i.e. vertices) and elements of *E* = (*e*_1_ *, e*_2_ *,…, e*_*m*_) are the edges, which are used to link the nodes. Instigating part *C*_*j*_ and reachable part *C*_*k*_ are respectively considered as start vertex *V*_0_ and target vertex *V*_target_. The intelligent parallel breadth-first algorithm is utilized to search all propagation paths from *V*_0_ to *V*_target_. At the same time, the change propagation probability between parts is recorded. Then a breadth-first tree can be obtained by intelligent parallel breadth-first algorithm, where *V*_0_ and *V*_target_ are respectively the root and top of the tree. This algorithm use the existing vertices and the edges between vertices in the process of searching paths from beginning to end, namely, the vertices closed to *V*_0_ are firstly searched and then the vertices far away from *V*_0_ are searched (Buluc and Madduri, 2011; Beamer et al., 2012; Garcia et al., 2020; Yigit et al., 2021). In search process, the vertices in current task queue form the boundary of accessed vertices, which is called active vertex set.

Assume *A* represents the adjacent matrix corresponding to directed graph *G*. If *a*_*ij*_ in *A* equals to 0, it means that there is no link from vertex *i* to vertex *j*. If *a*_*ij*_ equals to 1, it means that there is a link from vertex *i* to vertex *j*. We utilize *k* processors to parallel search paths. That is, matrix *A* is divided into *k* blocks, and every processor ***K*** (*p*, *q*) (1 ≤ *p* ≤ *m*/*k*, 1 ≤ *q* ≤ *m*/*k*) deals with a corresponding sub-matrix ***A***_*pq*_.

The search process of change propagation path from *V*_0_ to *V*_target_ based on intelligent parallel breadth-first algorithm is illustrated as follows.

Step 1: both the current boundary *B*_*ij*_ and the next boundary *N*_*ij*_ of breadth-first search algorithm are set to *null*, and the number of the lever of all the vertices is set to 0;
Step 2: all source nodes in graph *G* are input within the boundary corresponding to the processor where source nodes are located, which means that this vertex is reachable.
Step 3: each processor processes the assigned boundary in parallel. If the number of the lever in which a vertex of *B*_*ij*_ is located is 0, then it will be put into the temporary set *H*_*pq*_. Meantime, the number of the lever of this vertex adds 1 (initial value is set to 1). Then, the nodes of next boundary will be calculated and saved to *N*_*ij*_.
Step 4: After obtaining the set of the nodes of the lower boundary, they will be deemed as current boundary of the processor, and the number of the lever adds 1.
Step 5: if the number of the nodes in current boundary that need to be computed in the next time does not equal to 0, then go to step 3 and step 4. Otherwise, go to step 6.
Step 6: incorporate the lever number of all vertices in the processor, and get the information of the lever number of directed graph.
Step 7: get the information of all reachable paths of the vertices according to the layer number of the vertices.

### Change Impact Degree Analysis for Feeding Part Within the Module

Change propagation patterns between parts have two types: 1) one part passes the change onto the directly connected parts, for instance, from *C*_2_ to *C*_3_ and *C*_4_ as shown in Figure 2; and 2) change propagation from one part to another part through one or more intermediate parts, for instance, from *C*_2_ to *C*_4_ through intermediate part *C*_3_, from *C*_2_ to *C*_1_ through intermediate parts *C*_4_ and *C*_3_, both of them are indirect change propagation. There is not only direct change impact between some parts, but also indirect change impact. For some parts, there may be only indirect change impact. For example, there is no direct change impact of *C*_5_ on *C*_8_, since the change of *C*_5_ propagates to *C*_8_ through parts *C*_6_ and *C*_7_. The propagation paths of indirect change impact are all possible paths of change propagation divergence.

**FIGURE 4:**
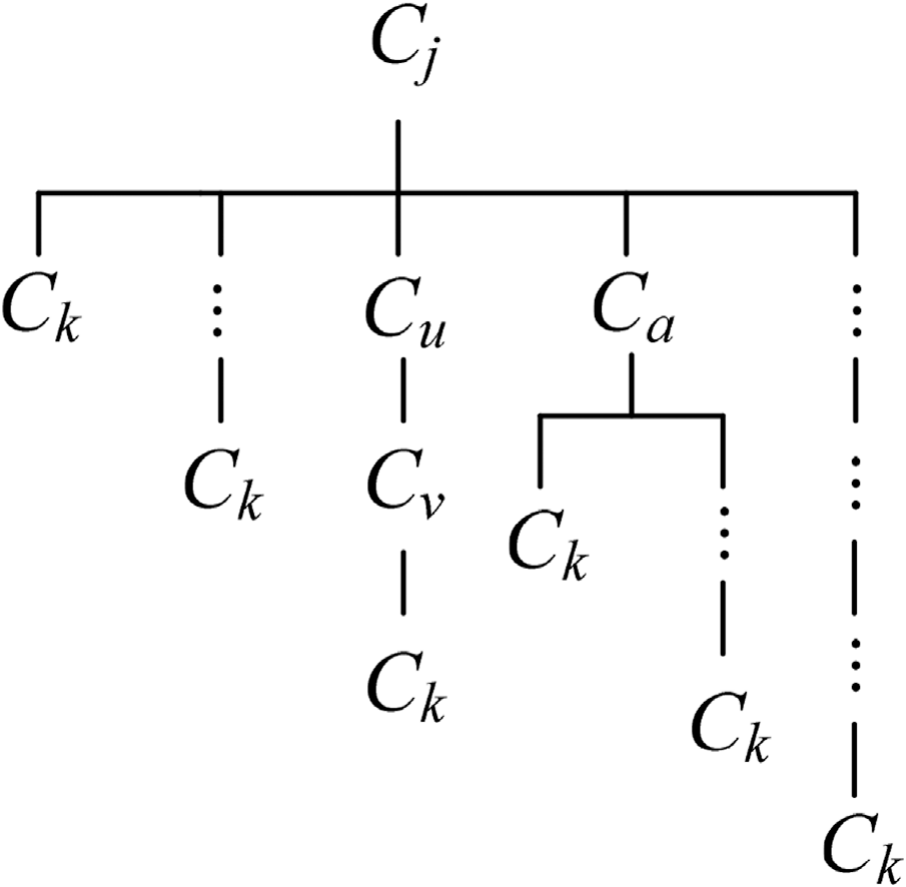
Change propagation tree.

The complex association relationships between parts make change propagation diffuse in different directions, which will form many change propagation paths. According to the relationships between parts and change propagation patterns, all direct and indirect CPPs from feeding part to reachable part can be identified, and then the change propagation tree is constructed, as show in Figure 4. The change propagation tree describes propagation characteristic of feeding part propagating within the module, and its every branch represents a direct or indirect path of change propagation. The elements within any path are not repeated, so the number of the elements in the longest path will not be more than the number of the parts within corresponding module.

Generally speaking, the closer the relationships among parts within the module, the greater the divergence of change propagation, and the more the branches of the change propagation tree. For a CPP, the probability of change impact is related to the length of this propagation path, i.e., the number of relevant parts. For the sake of simplicity, the term “(*C*_*j*_)→(*C*_*k*_)” is used to refer to direct CPP. Assume there exist distinct CPPs from part *C*_*j*_ to part *C*_*k*_, where *C*_*j*_→*C*_*k*_ is one direct path with the propagation probability of *r*_*k*,__*j*_. Of course, there may be no direct path, for instance, *C*_7_→*C*_5_, *r*_5_,_7_ = 0, as shown in Figure 1. The others *s*-1 paths are indirect. If *C*_*i*_→*C*_*u*_→*C*_*v*_→*C*_*k*_ is one of the indirect CPPs (the *r*th path), as shown in Figure 4, then the change impact degree of this path is expressed as follows:

$$p\left(C_{j},C_{kr}\right)=r_{u,j}\times r_{v,u}\times r_{k,v}$$ (4)

The different paths have different change impacts. All possible change impacts are reflected by corresponding propagation paths. The sum of change impacts of all possible CPPs from one part to another part are the change impact degree of the former on the latter. Hence, the change impact degree propagating from part *C*_*j*_ to part *C*_*k*_, *P*(*C*_*j*_, *C*_*k*_), is the sum of change impact degree of all of *s* distinct change propagation paths, namely

$$P\left(C_{j},C_{k}\right)=\sum_{r=1}^{s}P\left(C_{j},C_{kr}\right)$$ (5)

**FIGURE 5:**
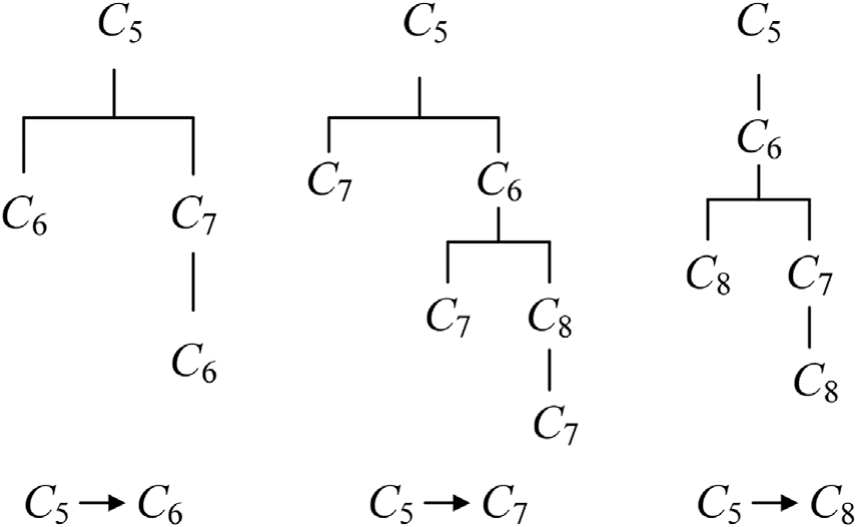
Change propagation tree from *C*_5_ to *C*_6_, *C*_7_ and *C*_8_.

Equation 5 can be applied to compute the change impact degree of the feeding part *C*_*j*_ on the reachable part *C*_*k*_. For instance, let us consider a case shown in Figure 1. The change of *C*_3_ in module *M*_1_ will propagate to *C*_5_ in module *M*_2_, and the latter will cause the alteration of *C*_6_, *C*_7_ and *C*_8_. Consequently, *C*_5_ can be regarded as feeding part of design change in *M*_2_, The modification of *C*_5_ will directly propagate to *C*_6_ and *C*_7,_ and the change of *C*_6_ will propagate to *C*_8_, whereby *C*_6_ is intercoupled with *C*_7_ that is intercoupled with *C*_8_. To analyze change impact propagation from *C*_5_ to all of other parts belong to *M*_2_, one should identify CPPs of *C*_5_ within *M*_2_. On basis of DDM in Figure 1 and change propagation network in Figure 2, the change propagation trees from *C*_5_ to *C*_6_, *C*_7_ and *C*_8_ in module *M*_2_ are constructed, as shown in Figure 5.

Then, according to change propagation trees in Figure 5, we can respectively compute change impact degrees of *C*_5_ on *C*_6_, *C*_7_ and *C*_8_, as following.

For *C*_5_ to *C*_6_: *P*(*C*_5_,*C*_6_) = *r*_6, 5_ + *r*_7, 5_ × *r*_6, 7_ = 0.6 + 0.5×0.1 = 0.65.

For *C*_5_ to *C*_7_: *P*(*C*_5_,*C*_7_) = 0.5 + 0.6 ×(0.4 + 0.4×0.3) = 0.846.

For *C*_5_ to *C*_8_: *P*(*C*_5_,*C*_8_) = 0.6 ×(0.4 + 0.4×0.6) = 0.384.

The above change impact degrees are added, and the result is comprehensive change impact degree of *C*_5_ on all of other parts in *M*_2_, i.e.

$$P\left(C_{5},M_{2}\right)=P\left(C_{5},C_{6}\right)+P\left(C_{5},C_{7}\right)+P\left(C_{5},C_{8}\right)=1.846$$

Module *M*_1_ has only one instigating *C*_3_ that affects *M*_2_, moreover, *M*_2_ has only one affected part *C*_5_, so the change impact degree of *M*_1_ on *M*_2_ is equal to the following.

$$P\left(M_{1},M_{2}\right)=P\left(C_{3},M_{2}\right)=r_{\mathrm{5,3}}\times P\left(C_{5},M_{2}\right)=\text{0}\text{.}2\times \left[1+1.846\right]=0.5692$$

## Identification of Influential Modules Considering Design Change Impacts

### Analysis on the Influence of Affected Part Within Module

As mentioned in *Analysis on Change* I*mpact Degree Between Modules*, the change propagation between modules is not only dependent on the association elements between modules, but also relies on the relationships among parts within the affected module and the influence of affected parts in the corresponding module in which it is located. For instance, module *M*_1_ affects part *C*_5_ in module *M*_2_, and module *M*_2_ affects part *C*_2_ in module *M*_1_, as shown in Figure 1. *C*_5_ is considered feeding part of change propagation from module *M*_1_ to module *M*_2_. Similarly, *C*_2_ is feeding part propagating from module *M*_2_ to module *M*_1_. The number of the parts for the two modules is the same. Their change propagation networks within modules *M*_1_ and *M*_2_ are similar and direct change probabilities are also near. Since the influence of part *C*_2_ in module *M*_1_ and part *C*_5_ in module *M*_2_ is not the same, their change impact degree is also different.

For each module, we can take any part as a change source, and then calculate its change impact degree that propagates to all of the other parts within the same module in which it is located. Assume a module *M* is composed of *m* parts, the change impact degree of the *p*th part within a module, *P*(*C*_*p*_, *M*), is expressed as follows.

$$P\left(C_{p},M\right)=\sum_{k=1,k\neq p}^{m}P\left(C_{p},C_{k}\right)$$ (6)

From the perspective of engineering change, the larger the value of change impact degree, the higher the influence of the corresponding part inside the module. According to change impact degree of each part, one can judge their influence within the same module. If the change impact degree of one part is the largest in the corresponding module, it means that this part has more significant effect on other parts. Namely, its influence is the highest in this module. So, in the design process of the modular products, it should be avoided as an affected part between modules as much as possible to prevent it from propagating further. If the change impact degree of one part is the smallest in the corresponding module, it means that this part has the smallest effect on other parts. When its value is equal to 0, it means that the corresponding part does not affect other parts and may limit the diffusion of change propagation.

For example, consider the example proposed change impacts discussed in *Analysis on Change Impact Degree Between Modules*. The change impact degrees of all parts in *M*_1_ and *M*_2_ are respectively shown in Table 1, 2.

**TABLE 1:** The change impact degrees of parts within *M*_1_.

*C*	1	2	3	4
*P*(*C*_*i*_, *C*_1_)	-	0.104	0.2	0.04
*P*(*C*_*i*_, *C*_2_)	0.838	-	0.44	0.088
*P*(*C*_*i*_, *C*_3_)	0.817	0.502	-	0.2
*P*(*C*_*i*_, *C*_4_)	0.832	0.2	0.704	-
*P*(*C*_*i*_, *M*_1_)	2.487	0.806	1.344	0.328
Priority of the influence	1	3	2	4

**TABLE 2:** The change impact degrees of parts within *M*_2_.

*C*	5	6	7	8
*P*(*C*_*i*_, *C*_5_)	-	0.156	0.3	0.09
*P*(*C*_*i*_, *C*_6_)	0.65	-	0.25	0.075
*P*(*C*_*i*_, *C*_7_)	0.846	0.52	-	0.3
*P*(*C*_*i*_, *C*_8_)	0.384	0.64	0.7	-
*P*(*C*_*i*_, *M*_2_)	1.846	1.316	1.25	0.465
Priority of the influence	1	2	3	4

It is evident from Table 1, 2 that part *C*_1_ has the largest effect on other parts (*P*(*C*_*i*_, *M*_1_) = 2.487) in *M*_1_. So *C*_1_ is a dominant part in *M*_1_, next is *C*_3_, and then is *C*_2_ and *C*_4_. Similarly, the priority of change impact in *M*_2_ is *C*_5_, *C*_6_, *C*_7_, and *C*_8_ in turn.

### Identification of Influential Modules

The change impact degree between any two modules embodies their direct influence, but it does not reflect the influence of design change for a module in the whole product. According to the definition from reference (Li et al., 2019), the influential modules are the modules that once the design changes, the cascading influence will be hard to control. In other words, the influential modules are those that have a significant impact on change propagation. So it is also necessary to analyze the impact of module changes from the perspective of the whole product, and consider simultaneously indirect change impacts between modules. Here, any a module is considered change source. We can analyse the influence of its instigating parts on other modules, and calculate its comprehensive change impact degree within a given product. According to comprehensive change impact degree of each module we can identify influential modules and the redesign priority of modules in products.

In *Identify Reachable Parts for Change Ppropagation*, we determined all reachable parts to which a change source propagated with reachable matrix. Consequently, in the process of analysing comprehensive change impact degree of module, the reachable matrix can be also used to identify reachable parts of other modules to which a change has occurred in the instigating part propagates. Then the change propagation network between modules is constructed according to the dependence between association parts. Take any module as change source and the corresponding instigating parts that affect other modules are determined. The CPPs of each instigating part are analyzed and captured, and the influence of each propagation path is discussed. The change impact degree of each instigating part on all reachable parts in other modules is computed. Here, other affected modules can be thought as an integrated module. The comprehensive change impact degree of upstream module in which instigating parts are located will be equivalent to direct change impact degree of upstream module on this integrated module.

**FIGURE 6:**
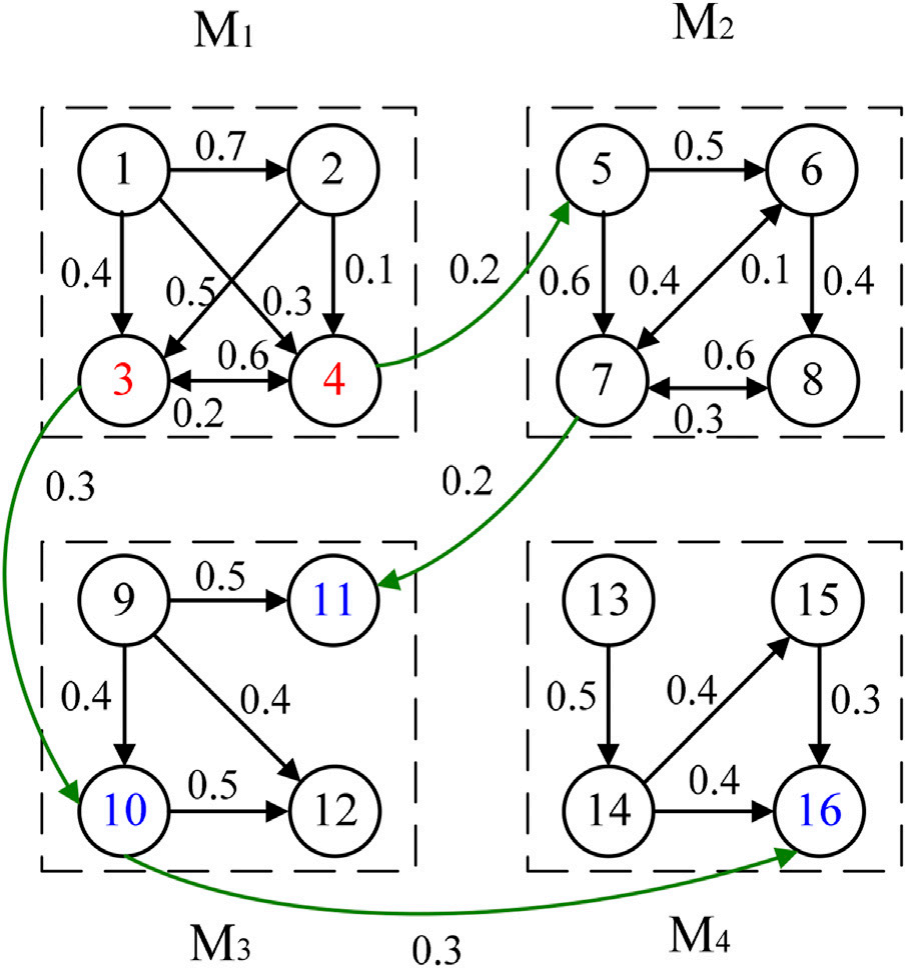
Change propagation network between modules.

Let us consider a product that consists of four modules. The change propagation network between modules is represented as Figure 6. The change of module *M*_1_ will directly cause alternation of modules *M*_2_ and *M*_3_. The modification of *M*_2_ will propagate to *M*_3_, and the latter simultaneously passes the change onto module *M*_4_. The measures, change impact degrees of parts within module used in *Analysis on the Influence of Affected Part within Module*, cannot be used directly to quantify comprehensive change impact degree of modules for the problem considered in this section, because their propagation ways are different. One module affects the other, but it does not mean that all parts of the former have an impact on the latter. Consequently, the module cannot be thought as node in change propagation network. For instance, *M*_2_ influences *M*_3_ and the latter influences *M*_4_ in Figure 6, but *M*_2_ does not affects *M*_4_. Since part *C*_11_ in *M*_3_ influenced by *M*_2_ is an absorption part, its change does not propagate to other parts. When the comprehensive change impact degree of *M*_1_ is computed, all of other modules, namely, *M*_2_, *M*_3_ and *M*_4_, can be regarded as an integrated module *M*_1_’. At this time, *C*_3_ and *C*_4_ are instigating parts. The change impact degree of *M*_1_ to *M*_1_’, is comprehensive change impact degree of *M*_1_ within the product, *P* (*M*_1_, *A*), i.e.

P(M1, A)= P(M1, M1′)= P(M1, M2) + P(M1, M3) + P(C4, C7)·P(C7, M3) + P(C3, C10)·P(C10, M4) (7)

where “*P* (*M*_1_, *M*_2_) + *P* (*M*_1_, *M*_3_)" is direct change impact degree of *M*_1_ on *M*_2_ and *M*_3_, “*P*(*C*_4_, *C*_7_)·*P*(*C*_7_, *M*_3_)" and “*P*(*C*_3_, *C*_10_)·*P*(*C*_10_, *M*_4_)" are indirect change impact degree of *M*_1_ on *M*_3_ and *M*_4_, respectively.

Assume a product is composed of *N* modules and *n* parts, where module *M*_*i*_ has *r* instigating parts that affect other modules, the comprehensive change impact degree of *M*_*i*_, *P* (*M*_*i*_, *A*), is expressed as

$$P\left(M_{i},A\right)=\sum_{j=1}^{r}P\left(M_{i},{M^{\prime }}_{i}\right)$$ (8)

where *P* (*M*_*i*_, *M*_*i*_’) is change impact degree of one instigating part in module *M*_*i*_ on all of other modules.

The larger the value of *P* (*M*_*i*_, *A*), the larger the influence of change propagation of module *M*_*i*_ within product. According to the value of *P* (*M*_*i*_, *A*) of each module, the influential modules can be identified.

Another method computing comprehensive change impact degree is based on the level of CPPs. Since the number of modules in the longest path is not more than *N*, the maximum level of propagation paths does not exceed *N* -1. Then we can calculate comprehensive change impact degree of any a module for each level. The first level is used to compute direct change impact degree of initial module. The second level is applicable to compute its indirect change impact degree that propagates to another module through one intermediate module that has a direct effect on the former,and so on. The last level is for indirect change impact degree through *N* -1 intermediate modules. The comprehensive change impact degree, *P* (*M*_*i*_, *A*) can be also represented as

$$\begin{array}{l} P\left(M_{i},A\right)=\sum_{j=1,j\neq i}^{N}P\left(M_{i},M_{j}\right)+\sum_{\begin{array}{l} k=1\\ k\neq j\neq i \end{array}}^{N}r_{b,a}\times P\left(C_{b},C_{c}\right)\times P\left(C_{c},M_{k}\right)+\cdots \\ +\sum_{\begin{array}{l} p=1\\ p\neq j\neq \cdots \neq i \end{array}}^{N}r_{b,a}\times P\left(C_{b},C_{c}\right)\times r_{d,c}\times P\left(C_{d},C_{e}\right)\times \cdots \times r_{v,u}\times P\left(C_{v},C_{w}\right)\times P\left(C_{w},M_{p}\right) \end{array}$$ (9)

where $\sum_{j=1,j\neq i}^{N}P\left(M_{i},M_{j}\right)$ is direct impact degree of initial module *M*_*i*_ on other modules in the first level of propagation paths; $\sum_{\begin{array}{l} k=1\\ k\neq j\neq i \end{array}}^{N}r_{b,a}\times P\left(C_{b},C_{c}\right)\times P\left(C_{c},M_{k}\right)$ is indirect impact degree of *M*_*i*_ in the second level of the path, whereas *M*_*i*_ affects indirectly *M*_*k*_ through intermediate *M*_*j*_ that has a direct influence on *M*_*k*_. Both parts *C*_*b*_ and *C*_*c*_ belong to *M*_*j*_ that is directly affected by *M*_*i*_. *r*_*b*,__*a*_ is the probability of change propagation from part *C*_*a*_ in *M*_*i*_ to *C*_*b*_, *C*_*b*_ is affected part to *M*_*i*_, and *C*_*c*_ is an instigating part to *M*_*k*_. The last item in Eq. 9 is indirect impact degree of *M*_*i*_ in the last level of the path, whereas *r*_*d*,__*c*_, … , *r*_*v*,__*u*_ are respectively change probability from instigating parts to affected parts between association modules, *P*(*C*_*w*_, *M*_*p*_) is change impact degree of part *C*_*w*_ that belongs to the previous module to *M*_*p*_, and *P*(*C*_*d*_, *C*_*e*_),…, *P*(*C*_*v*_, *C*_*w*_) are respectively change impact degree within modules from feeding part to instigating part that affects the next module in the path.

### Solution to Comprehensive Change Impact Degree Based on Bat Algorithm

With the increase of the number of parts, the number of propagation paths will increase exponentially. The comprehensive change impact degree involves massive calculation and is time-consuming. The biological evolution algorithms have been widely used (Li and Yin, 20112011a; Li and Yin, 20112011b; Chen et al., 2021a; Chen et al., 2021b). The bat algorithm has very high efficiency in optimization calculation (Mashwani et al., 2021; Saji and Barkatou, 2021). So this article uses bat algorithm to solve comprehensive change impact degree of complex network model.

#### The Principle of Bat Algorithm

Bat algorithm has the following three idealized assumptions (Yang and He, 2013; Xu and Zhang, 2016).

1) All bats use echolocation to perceive distance, and can distinguish the food or prey and obstacles in the background in the way we do not know.
2) At first, the bat flies at random speed and direction. They can search the prey through changing the wavelength *λ* and loudness *S.* Simultaneously, they can automatically adjust the transmitted pulse wavelength and rate *r* (∈[0, 1]) according to the distance.
3) Assume that the loudness varies gradually from the maximum value *S* (1) to the lowest constant value *S*_min_.

#### Solution to Bat Movement

We will input the bats into *n*-dimension space. The frequency of the *i*th bat is *F*_*i*_, and its position and speed are respectively, *x*_*i*_ = (*x*_*i*1_, *x*_*i*2_, … , *x*_*in*_) and *v*_*i*_=(*v*_*i*1_, *v*_*i*2_, … , *v*_*in*_). Then the equations of the *t* generation for the bat *i* on the position and speed are as follows.

$$F_{i}=F_{\min}+\left(F_{\max}-F_{\min}\right)\beta \beta \in \left[0,1\right]$$ (10)

$$v_{id}\left(t+1\right)=v_{id}\left(t\right)+F_{i}\left(v_{id}\left(t\right)-p_{gd}\left(t\right)\right)$$ (11)

$$x_{id}\left(t+1\right)=x_{id}\left(t\right)+v_{id}\left(t+1\right)$$ (12)

where, *F*_min_ and *F*_max_ are respectively the maximum and minimum frequencies, *β*∈[0, one] is a random vector and its elements obey uniform distribution; *x*_*id*_ (*t*) and *x*_*id*_ (*t* +1) are respectively the *d*th-dimension position of bat *i* in the *t*th and (*t*+1)-th iteration optimization process, *v*_*id*_ (*t*) and *v*_*id*_ (*t*+1) are their corresponding speeds, and *p*_*gd*_ (*t*) is the *d*th-dimension position of bat *g* with optimal global fitness in the *t*th and (*t*+1)-th iteration optimization process.

When the bat performs a global search, it also conducts a local refinement search to find a better solution. Once a best solution *x*_*old*_ is chosen randomly from the current optimal solution set, the new pending position of each bat is generated nearby, as shown in type (13).

$$x_{new}=x_{old}+\varepsilon S\left(t\right)$$ (13)

where, *ε*∈ (Bonvoisin et al., 2016Bonvoisin et al., 2016) is an arbitrary number, *S*(*t*) is the average loudness of all bats in the *t*th iteration.

In addition, the loudness *S*_*i*_ and rate *r*_*i*_ of pulse emission, are required to update with iterative process, in order to achieve a good balance of the algorithm between global search and local search. Update equation is as follows.

$$S_{i}\left(t+1\right)=\alpha S_{i}\left(t\right)$$ (14)

$$r_{i}\left(t+1\right)=r_{i}\left(1\right)\left[1-\exp\left(-\gamma \left(t+1\right)\right)\right]$$ (15)

where, *α* is loudness attenuation factor and is a constant, 0<*α* < 1; *γ* is pulse frequency increase coefficient and is also a constant, *γ* > 0; *S*_*i*_ (*t*) and *S*_*i*_ (*t*+1) are respectively the loudness of bat *i* in the *t*th and (*t*+1)-th iteration optimization process; *r*_*i*_ (*t*) and *r*_*i*_ (*t*+1) are respectively the pulse rate of bat *i* in the *t*th and (*t*+1)-th iteration optimization process.

Assume the size of the bat population is *n*, and the position of the *i*th bat is *x*(*i*)*.* The steps of solving the largest comprehensive change impact degree using the bat algorithm is given as follows.

Step 1. Build the network model of the product parts, and set parameters’ values of bat algorithm, such as the size of the bat population, the maximum number of iterations, and so on.
Step 2: Initialize the position *x*(*i*), speed *v*(*i*), frequency *F*(*i*), the pulse emission rate *r*_*i*_ (1), and the pulse loudness *S*_*i*_(1). Apply bat algorithm to calculate the local optimal value, *P*(*M*_*i*_^*’*^, *A*), and find out the corresponding optimal bat individual in the population.
Step 3: According to Eqs 10–12, update the position and speed of the bat in the iteration process.
Step 4: Produce a random number *rand*. If *rand* > *r*_*i*_ (*t*), a new local solution *P* ^*'*^(*M*_*i*_^*’*^, *A*) is obtained by Eq. 13.
Step 5: For each bat individual, the corresponding random number *rand* is created. If *rand* < *S*_*i*_ and *P* ^*'*^(*M*_*i*_^*’*^, *A*)>*P*(*M*_*i*_^*’*^, *A*), then this solution is accepted. At the same time *r*_*i*_(*t*) and *S*_*i*_ (*t*) are updated by Eqs 14, 15.
Step 6: Judge whether stop conditions are met. If they are satisfied, turn to the next step. Otherwise, go to step 3.
Step 7: Update and output the global optimal solution.

## Case Study

**FIGURE 7:**
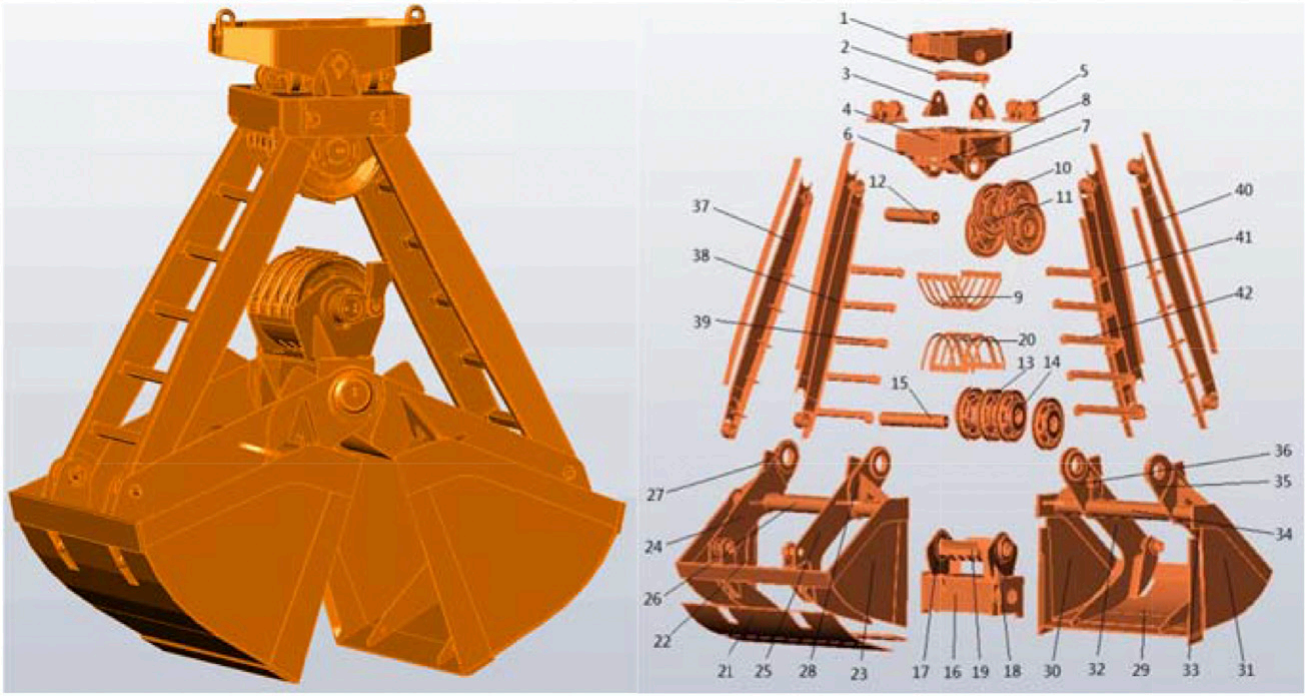
Structure of the grab and its components explosion.

In this section, a case study on the crane grab is provided to illustrate the proposed analysis method of change propagation between modules and to identify influential modules. The grab is a kind of the special load handling device for the crane, which is mainly used to grab bulk cargoes. A typical crane grab contains 42 key components. Figure 7 illustrates the structure of the grab and its components explosion, and Table 3 summarizes a list of its key components. Cheng et al. (Cheng et al., 2018) clustered the components of the grab into nine modules using DBSCAN algorithm. The name of each module and the serial number of the components it contains are shown in Table 4.

**TABLE 3:** List of key components of the grab.

No	Name	No	Name	No	Name
1	Balance frame	15	Lower pulley shaft	29	Right grab bucket floor
2	Balancing shaft	16	Lower bearing beam	30	Right grab side plate 1
3	Balance frame base	17	Side plate 1 of lower bearing beam pulley	31	Right grab side plate 2
4	Upper bearing beam	18	Side plate 2 of lower bearing beam pulley	32	Right grab bent plate 1
5	Guide rope device	19	Lower bearing beam pulley cover plate	33	Right grab bent plate 2
6	Side plate 1 of upper bearing beam pulley	20	Lower protective frame	34	Right grab pipe
7	Side plate 2 of upper bearing beam pulley	21	Left grab bucket floor	35	Right grab center ear plate 1
8	Upper bearing beam pulley cover plate	22	Left grab side plate 1	36	Right grab center ear plate 2
9	Upper protective frame	23	Left grab side plate2	37	Left strut 1
10	Upper pulley group	24	Left grab bent plate 1	38	Left strut 2
11	Bearing group of upper pulley group	25	Left grab bent plate 2	39	Left strut square tube
12	Upper pulley shaft	26	Left grab pipe	40	Right strut 1
13	Lower pulley group	27	Left grab center ear plate 1	41	Right strut 2
14	Bearing group of lower pulley group	28	Left grab center ear plate 2	42	Right strut square tube

**TABLE 4:** The name of each module and the serial number of the components it contains.

No	Name	The serial number of the components
*M*_1_	Lower pulley group	(13, 15, 14)
*M*_2_	Upper pulley group	(10, 12, 11)
*M*_3_	Upper bearing beam	(4, 6, 7, 8, 9, 1, 2, 3)
*M*_4_	Guide rope device	(5)
*M*_5_	Lower bearing beam	(16, 17, 18, 19, 20)
*M*_6_	Left grab bucket body	(21, 22, 23, 24,25, 26, 27, 28)
*M*_7_	Right rab bucket body	(29, 30, 31, 32, 33, 34, 35, 36)
*M*_8_	Left strut	{37, 38, 39}
*M*_9_	Right strut	{40, 41, 42}

**FIGURE 8:**
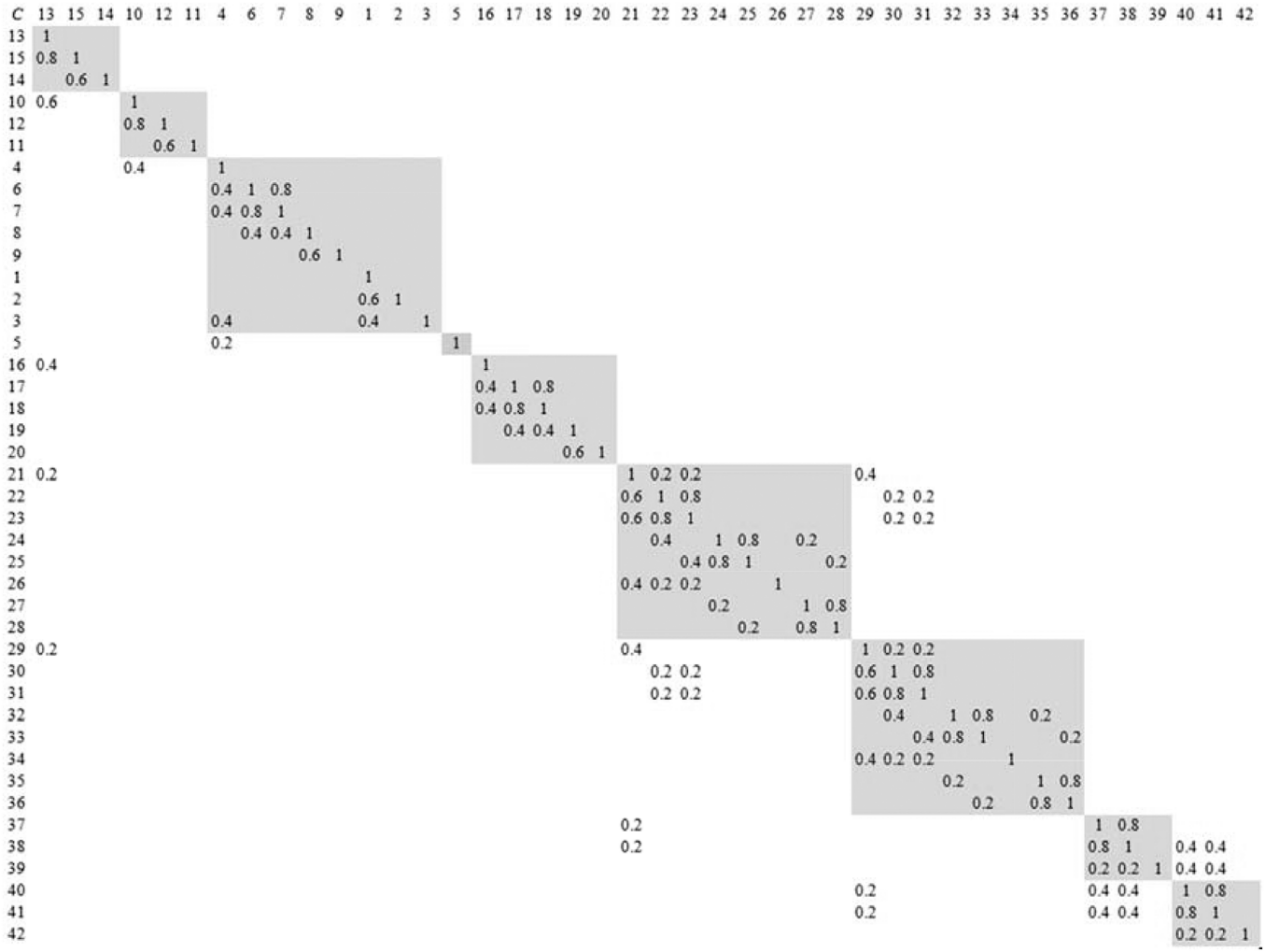
DDM of the grab with module.

**FIGURE 9:**
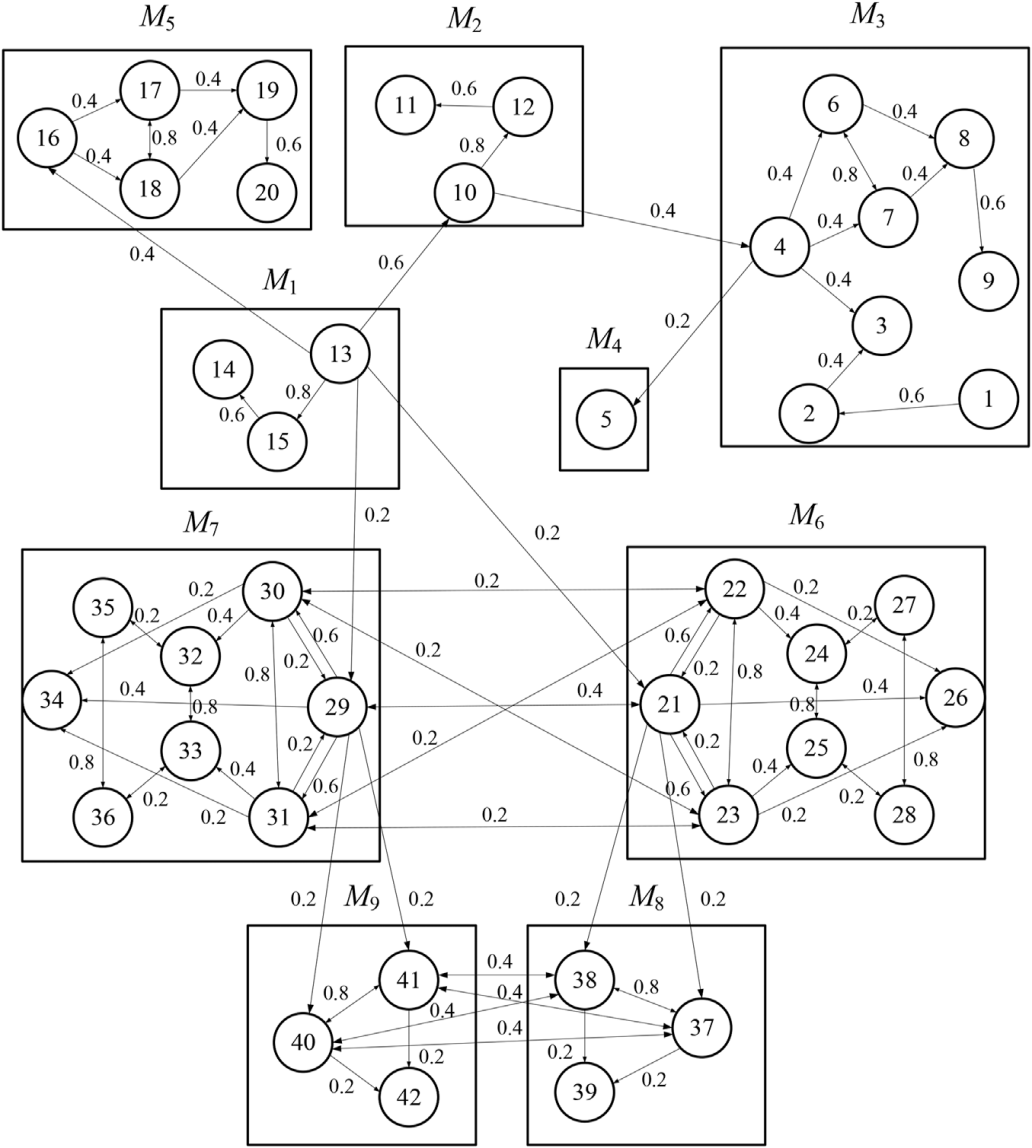
Change propagation network for the grab.

According to dependence relationships between components of the grab, the probability of change propagation from one part to another part is analysed and identified, and DDM of its modules is constructed, as shown in Figure 8. The change propagation network that represents design change relationships among modules and parts in the modules is constructed, as shown in Figure 9.

**FIGURE 10:**
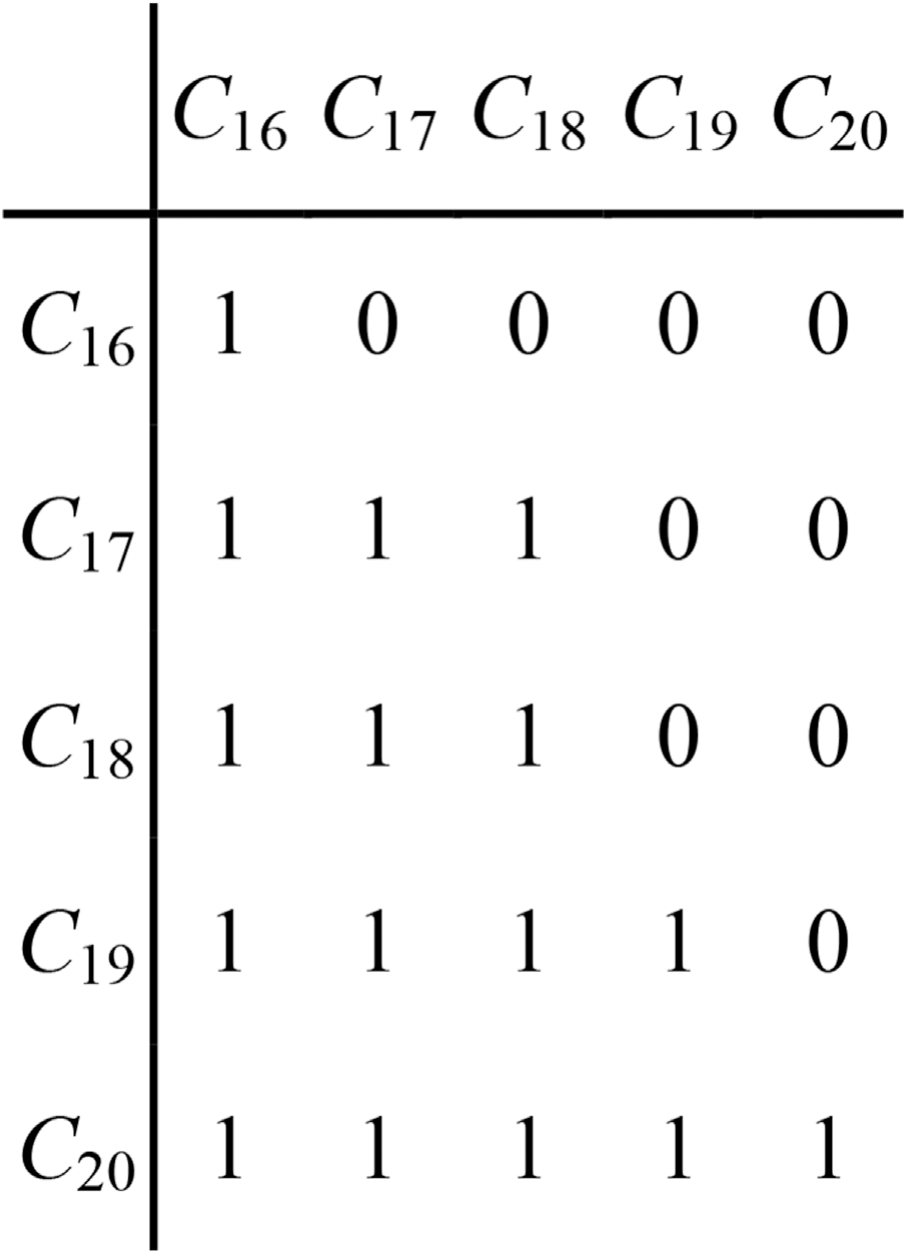
Reachable matrix of *M*_5_ for grab.

Then, the change impact degree between modules can be calculated according to the proposed method. The influence of *M*_1_ on *M*_5_ is taken as an example to describe and analyse change propagation from the former to the latter. It can be seen from Figures 8, 9 that *M*_1_ passes the change of part *C*_13_ onto the directly connected parts *C*_16_ in *M*_5_, whereby the change of *C*_16_ will propagate to other parts, such as parts *C*_17_, *C*_18_, and so on. So, *C*_16_ is deemed as a feeding part of change propagation in *M*_5_. To analyse change propagation of *C*_16_, the reachable matrix of *M*_5_ is firstly computed, and the result is shown as Figure 10. It is evident from Figure 10 that the reachable parts of change propagation for *C*_16_ include not only *C*_17_ and *C*_18_ (direct propagation) but also *C*_19_ and *C*_20_ (indirect propagation), i.e., the modification of *C*_16_ will lead to the alteration of all of other parts in *M*_5_.

**FIGURE 11:**
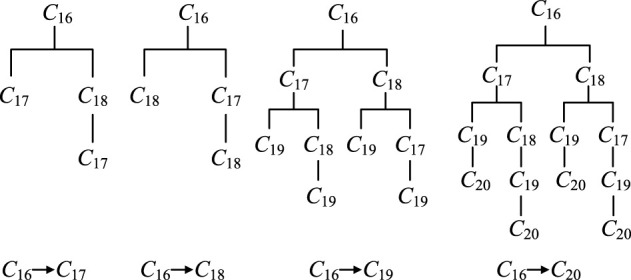
Change propagation tree of *C*_16_ in *M*_5_.

Next, the change propagation trees from *C*_16_ to *C*_17_, *C*_18_, *C*_19_ and *C*_20_ in *M*_5_ are constructed through parallel breadth-first search algorithm, as shown in Figure 11. All possible propagation paths of *C*_16_ within *M*_5_ are captured. The change impact degree of *C*_16_ on each reachable part is calculated as following.

For *C*_16_ to *C*_17_, it has two propagation paths, one is direct path from *C*_16_ to *C*_17_, and another is indirect path from *C*_16_ to *C*_17_ through intermediate part *C*_18_. So the change impact degree of *C*_16_ on *C*_17_ is.

*P*(*C*_16_, *C*_17_) = *r*_17, 16_ + *r*_18, 16_ × *r*_17, 18_ = 0.4 + 0.4 × 0.8 = 0.72.

For *C*_16_ to *C*_18_, *P*(*C*_16_, *C*_18_) = 0.4 + 0.4 × 0.8 = 0.72.

For *C*_16_ to *C*_19_, *P*(*C*_16_, *C*_19_) = 0.576.

For *C*_16_ to *C*_20_, *P*(*C*_16_, *C*_20_) = 0.3456.

The sum of above change impact degrees propagating from *C*_16_ to *C*_17_, *C*_18_, *C*_19_ and *C*_20_, is comprehensive change impact degree of *C*_16_ on all of other parts within *M*_5_, i.e.

*P*(*C*_16_, *M*_5_) = *P*(*C*_16_, *C*_17_) + *P*(*C*_16_, *C*_18_) + *P*(*C*_16_, *C*_19_)+ *P*(*C*_16_, *C*_20_) = 2.3616.

*M*_1_ has only one instigating part *C*_13_ that has effect on *M*_5_ and just influence *C*_16_ of *M*_5_, so the change impact degree of *M*_1_ on *M*_5_ is following.

*P* (M_1_, M_5_) = 0.4×[1 + 2.3616] = 1.34464.

Similarly, the change impact degrees between other modules can be computed, and then the comprehensive impact degrees of all modules can be obtained by bat algorithm. The results are shown as Table 5.

**TABLE 5:** The results of change impact degrees of modules.

	*M*_1_	*M*_2_	*M*_3_	*M*_4_	*M*_5_	*M*_6_	*M*_7_	*M*_8_	*M*_9_
*P* (*M*_*i*_, *M*_1_)	-	-	-	-	-	-	-	-	-
*P* (*M*_*i*_, *M*_2_)	1.368	-	-	-	-	-	-	-	-
*P* (*M*_*i*_, *M*_3_)	-	1.4125	-	-	-	-	-	-	-
*P* (*M*_*i*_, *M*_4_)	-	-	0.2	-	-	-	-	-	-
*P* (*M*_*i*_, *M*_5_)	1.3446	-	-	-	-	-	-	-	-
*P* (*M*_*i*_, *M*_6_)	1.1151	-	-	-	-	-	5.8281	-	-
*P* (*M*_*i*_, *M*_7_)	1.1151	-	-	-	-	5.8281	-	-	-
*P* (*M*_*i*_, *M*_8_)	-	-	-	-	-	0.864	-	-	3.456
*P* (*M*_*i*_, *M*_9_)	-	-	-	-	-	-	0.864	3.456	-
*P* (*M*_*i*_, *A*)	9.5863	1.4925	0.2	0	0	9.0867	9.0867	3.456	3.456

Table 5 shows the influence of change propagation for each module. The value of *P* (*M*_1_, *A*) is largest, followed by *P* (*M*_6_, *A*) and *P* (*M*_7_, *A*). Moreover, the comprehensive change impact degrees of these three modules are much bigger than that of other modules. So *M*_1_, *M*_6_, and *M*_7_ are influential modules in the grab. Both *P* (*M*_4_, *A*) and *P* (*M*_5_, *A*) equal to 0, which mean that *M*_4_ and *M*_5_ have no influence on other modules, in other words, both of them are full absorption modules. *M*_8_ and *M*_9_ have the same structure with synergistic functions. They just interact with each other, and have no effect on other modules. So they can be also regarded as absorption modules. From the standpoint of engineering design, design change of modules *M*_1_, *M*_6_, and *M*_7_ should be controlled as much as possible to effectively reduce the cost of product development and prevent the product failure.

## Conclusion

Product modularization has a significant influence on the product development process and the whole product lifecycle. There often exist association relationships between modules. A change of one part/module may cause other parts or modules to change, which in turn propagates through a product. The change between modules is not only dependent on the association element between modules, but also relies on the relationships among parts within an affected module and the influence of the affected part in the corresponding module in which it is located. The larger the size of the module and the stronger the influence of the affected part in the corresponding module, the greater the scope of change propagation.

The proposed approach measures the relative change impacts of modules and parts in the modules. All possible CPPs are determined by a parallel breadth-first search algorithm. A reachable matrix is employed to identify which parts are influenced directly or indirectly by the affected part within the corresponding module in which it is located. A change propagation network that represents design change relationships among parts and modules is constructed. Then the direct and indirect impacts of change propagation are integrated, and the relative change impact degrees of modules and parts in the modules are computed. The influential modules are identified by the bat algorithm from the perspective of engineering change. Finally, an application of the proposed methods of association analysis and change impacts is demonstrated by an example of the crane grab. The methodology is applied to the design of other modular products. The design change impact in the modular product families can be studied in the future.

## Data Availability

The raw data supporting the conclusion of this article will be made available by the authors, without undue reservation.
